# PET/CT with ^18^F-choline or ^18^F-FDG in Hepatocellular Carcinoma Submitted to ^90^Y-TARE: A Real-World Study

**DOI:** 10.3390/biomedicines10112996

**Published:** 2022-11-21

**Authors:** Luca Filippi, Oreste Bagni, Ermanno Notarianni, Adelchi Saltarelli, Cesare Ambrogi, Orazio Schillaci

**Affiliations:** 1 Nuclear Medicine Unit, “Santa Maria Goretti” Hospital, Via Antonio Canova, 04100 Latina, Italy; 2 Diagnostic and Interventional Unit, “Santa Maria Goretti” Hospital, Via Antonio Canova, 04100 Latina, Italy; 3 Department of Biomedicine and Prevention, University Tor Vergata, Viale Oxford 81, 00133 Rome, Italy

**Keywords:** positron emission tomography, molecular imaging, hepatocellular carcinoma, ^90^Y-microspheres, ^18^F-choline, ^18^F-FDG

## Abstract

Our aim was to assess the role of positron emission computed tomography (PET/CT) with ^18^F-choline (^18^F-FCH) or ^18^F-fluorodeoxyglucose (^18^F-FDG) in hepatocellular carcinoma (HCC) submitted to ^90^Y-radioembolization (^90^Y-TARE). We retrospectively analyzed clinical records of 21 HCC patients submitted to PET/CT with ^18^F-fluorocholine (^18^F-FCH) or ^18^F-fluodeoxyglucose (^18^F-FDG) before and 8 weeks after ^90^Y-TARE. On pre-treatment PET/CT, 13 subjects (61.9%) were ^18^F-FCH-positive, while 8 (38.1%) resulted ^18^F-FCH-negative and ^18^F-FDG-positive. At 8-weeks post ^90^Y-TARE PET/CT, 13 subjects showed partial metabolic response and 8 resulted non-responders, with a higher response rate among ^18^F-FCH-positive with respect to ^18^F-FDG-positive patients (i.e., 76.9% vs. 37.5%, *p* = 0.46). Post-treatment PET/CT influenced patients’ clinical management in 10 cases (47.6%); in 8 subjects it provided indication for a second ^90^Y-TARE targeting metabolically active HCC remnant, while in 2 patients it led to a PET-guided radiotherapy on metastatic nodes. By Kaplan–Meier analysis, patients’ age (≤69 y) and post ^90^Y-TARE PET/CT’s impact on clinical management significantly correlated with overall survival (OS). In Cox multivariate analysis, PET/CT’s impact on clinical management remained the only predictor of patients’ OS (*p* < 0.001). In our real-world study, PET/CT with ^18^F-FCH or ^18^F-FDG influenced clinical management and affected the final outcome for HCC patients treated with ^90^Y-TARE.

## 1. Introduction

Hepatocellular carcinoma (HCC) represents the fifth most common malignancy and a leading cause of cancer-related death worldwide [1]. Surgery is the therapy of choice for localized HCC, but the condition of some patients is not amenable to surgery, due to several reasons, such as large or multifocal lesions, portal vein invasion (PVI), extrahepatic spreading, poor liver function, etc. Advanced HCC has limited therapeutic options: tyrosine kinase inhibitors (TKIs), for example, proved effective in delaying disease progression; additionally, prolonging overall survival and immunotherapy with checkpoint inhibitors was found to exert strong anti-tumor activity in a subset of HCC patients [2,3].

Transarterial therapies, a group of treatments based on the intra-arterial administration of embolic or cytotoxic agents directed into the target lesions through their arterial feeders, play a crucial role in HCC therapeutic workflow [4]. In particular, the intra-arterial administration of glass or resin microspheres labeled with the radionuclide yttrium-90 (^90^Y), also known as selective internal radiation therapy (SIRT) or ^90^Y-transaterial radioembolization (^90^Y-TARE), has gained an ever-increasing importance for the management of liver cancer [5,6], demonstrating a satisfying response rate and relevant impact on patients’ quality of life [7]. In a meta-analysis including 21 studies investigating TARE’s impact on intermediate and advanced HCC, the pooled post-TARE overall survival (OS) resulted in 63% and 27% at 1- and 3-years, respectively, in intermediate stage HCC, while OS was 37% and 13% at the same time intervals in subjects with preserved liver function (Child-Pugh A-B7) but with an advanced HCC [8].

The optimal imaging modality to assess response to ^90^Y-TARE remains open to debate. ^90^Y-TARE, combining radiation therapy and embolization in a unique approach, can determine radiation-related changes in target lesions and the neighboring hepatic parenchyma. In such cases, response assessment with radiological techniques (computed tomography/CT or magnetic resonance imaging/MRI) might be challenging. HCC decrease in size after ^90^Y-TARE may occur relatively late (i.e., 4–6 months after the procedure) and persistent tumor contrast–enhancement or residual enhancing areas have been reported as common findings on early post-treatment imaging evaluation with CT or MRI [9]. Positron emission computed tomography (PET/CT) has a well-established role for tumor staging and monitoring after therapy, but its contribution in HCC treated with ^90^Y-TARE has not yet been extensively explored. ^18^F-FDG, the most commonly employed radiopharmaceutical for PET imaging, has been found useful to visualize only the most aggressive and less differentiated HCC forms, while the majority of well-differentiated HCCs do not incorporate this tracer due to several biological factors (i.e., low expression of glucose transporters/GLUT and relatively high level of glucose-6-phosphatase) [10]. ^18^F-labeled choline (^18^F-FCH), a surrogate biomarker of phospholipid biosynthesis, has been employed with good results for PET imaging of well-differentiated lesions [11], while a combined use of the two tracers (^18^F-FCH and ^18^F-FDG) has been proposed for the imaging of HCC according to the grade of tumor differentiation [12].

A recently published paper by Reizine et al. showed that the evaluation of response by ^18^F-FCH/^18^F-FDG PET/CT in HCC patients submitted to ^90^Y-TARE predicts 6-month response and their final outcome [13]. However, the impact of dual tracer PET imaging on patient management and, in particular, its potential to identify post ^90^Y-TARE metabolically active tumor remnant amenable to further ^90^Y-microsphere treatment has yet to be defined.

The aims of this retrospective study were: (1) to assess if post-treatment response assessed with ^18^F-FCH or ^18^F-FDG PET/CT influenced patients’ clinical management; (2) to determine whether the implementation of PET-directed therapies affected patient survival after ^90^Y-TARE.

## 2. Materials and Methods

### 2.1. Study Design

In this retrospective analysis, we included all the consecutive HCC patients who were examined on our PET/CT with ^18^F-FCH or, in case of ^18^F-FCH-negative tumors, with ^18^F-FDG before and 8 weeks after ^90^Y-TARE between 01/2018 and 03/2019. Previous medical history, including presence of cirrhosis, previously performed loco-regional or systemic therapies, the results of performed diagnostic imaging (contrast-enhanced CT, MRI, liver ultrasonography), and laboratory tests (e.g., alphafetoprotein, hepatic enzymes, bilirubin, albumin) were recorded. All of the patients included had to present a complete and detailed available clinical history.

Selected patients were then reviewed on a case-by-case basis and were identified as those belonging to 1 of these 2 possible clinical settings:

(a) HCC patients showing a pre-treatment ^18^F-FCH-positive PET/CT scan who were treated with ^90^Y-TARE and monitored with an 8-week post ^18^F-FCH PET/CT procedure;
(b) HCC patients with a pre-treatment ^18^F-FCH-negative and a ^18^F-FDG-positive PET/CT scan, who were submitted to ^90^Y-TARE and then followed-up with an 8-week ^18^F-FDG PET/CT scan.

The primary endpoint of the study was to define if an 8-week post-treatment response assessed by PET/CT with ^18^F-FCH or ^18^F-FDG influenced patients’ clinical management. PET/CT’s impact was scored as significant if (1) it provided an indication for a further ^90^Y-TARE procedure selectively targeting metabolically active HCC remnant detected on PET/CT imaging; (2) it entailed the implementation of PET-directed RT on isolated metastatic localizations. The secondary endpoint was to determine whether PET-directed therapies affected patients’ final outcome (i.e., OS). 

This was a retrospective study on data available for clinical practice in which clinical records of all patients in follow-up for HCC submitted to ^90^Y-TARE were reviewed. Data were anonymously collected and cumulatively gathered in an electronic database for analysis. Patients were not required to give informed consent to the study because the analysis used anonymous data that were obtained after each patient agreed to be followed-up and to collect clinical records by institutions. No experimental procedures, novel devices, or experimental drugs were used, and no founds were received. The study protocol conformed to the ethical guidelines of the 1975 Declaration of Helsinki.

### 2.2. ^90^Y-TARE Procedure

The enrollment criteria for ^90^Y-TARE were: previously diagnosed HCC (i.e., histologically proven or with imaging-based diagnosis); liver-only or liver-predominant disease; age ≥ 18 years; ability and willingness to provide written informed consent; life expectancy > 3 months; preserved liver function with Child–Pugh Class A or B (≤7 score); Eastern Cooperative Oncology Group (ECOG) performance status ≤ 2; bilirubin < 2.0 mg/dL, albumin > 2.0 g/dL, international normalized ratio (INR) < 1.5; creatinine < 2.0 mg/dL; platelets ≥ 100,000/μL, Hb ≥ 9.0 g/dL, and WBC ≥ 1500/μL. Patients with predominant extrahepatic disease, active CNS metastases, or diffuse peritoneal metastases were excluded [14].

All patients provided written informed consent prior to procedure and associated risk. Pre-procedural evaluation included baseline imaging studies (liver sonography, clinical and laboratory examination, ce-CT, and PET/CT).

Angiography with selective visceral catheterization was performed in order to evaluate the vascular and tumor anatomy and blood-flow dynamics. A ^99m^Tc-macroaggregated albumin scan was carried out to test gastrointestinal flow and to estimate the percent of injected activity shunted to the lungs. After 7–10 days, the patients returned to our department for a treatment session performed by selective catheterization of the main hepatic artery by the transfemoral approach, embolization of gastroduodenal and gastric artery. After selective catheterization of the right/left hepatic artery, the patient, without sedation, was administered with a slow, manually controlled injection lasting about 30 min, under intermittent fluoroscopic guidance, alternating the ^90^Y-microspheres suspended in 5% glucose solution with contrast medium for assessing persevered anterograde arterial flow. In all cases resin spheres (SIR-Spheres; Sirtex Medical, Sydney, Australia) were administered. In the case of bilobar lesions, each hepatic lobe was sequentially administered with ^90^Y-microspheres in a separate session within an interval of 6–8 weeks to reduce the risk of radioembolization-induced liver disease (RIELD).

The prescribed ^90^Y activity was determined as the patient-specific activity according to the body surface area (BSA) formula. After ^90^Y-TARE procedure, all subjects underwent a ^90^Y-PET/CT scan to assess the microsphere distribution pattern [15].

### 2.3. Imaging 

All patients underwent a PET/CT scan 20 min after the intravenous (i.v.) administration of 3.7 KBq/kg of ^18^F-methyl-choline (IASOcholine/Pcolina^®^, Iason GmbH, Graz Seiersberg, Austria) or 60 min after i.v. administration of 3.7 KBq/kg of ^18^F-FDG (Gluscan^®^, Advanced Accelerator Applications, Venafro, Italy) according to the International Guidelines [16]. For both radiopharmaceuticals, the PET/CT device was a Discovery ST (General Electric, GE, Milwaukee, WI, USA) with bismuth germanate crystal units arranged to form 24 rings combined with a 16-slice Light Speed Plus CT scanner. The average FWHM axial resolution of PET (full width at half maximum) is 5.2 mm and system sensitivity 9.3 cps/KBq for 3D acquisition mode. Scanning was performed from the neck to the proximal tight in 3D modality, with an acquisition time of 3 min per table position. Images were reconstructed by using an ordered subset expectation maximization iterative algorithm (OSEM-SV, VUE Point HD, GE, 2 iterations, 15 subsets). The CT was performed immediately before PET in the identical axial field of view using a standardized protocol consisting of automatic tube current modulation with auto mA-tube rotation time of 0.5 s/rotation, slice thickness of 3.75 mm. The CT data were resized from 512 × 512 to a 256 × 256 matrix to match the PET data. The data were transmitted to a nuclear medicine database, fused, and displayed using dedicated software (Advantage, GE).

### 2.4. Pre-Treatment Image Evaluation

Before ^90^Y-TARE, all patients underwent an ^18^F-FCH PET/CT scan as the first line PET diagnostic modality. Each ^18^F-FCH PET/CT scan was reviewed jointly by 2 board-certified nuclear medicine physicians (L.F. and O.B., both with >15 years of experience); images were visually evaluated for pathological tracer uptake, defined as a focally increased radiopharmaceutical’s incorporation within the hepatic lesions greater than that of the neighboring parenchyma, and were classified as positive or negative. In the case of negative ^18^F-FCH PET/CT scans, patients were submitted to ^18^F-FDG PET/CT scan that was carried out within 1 week from previously performed ^18^F-FCH imaging. HCCs were considered as ^18^F-FDG-positive if they showed increased tracer uptake greater than adjacent normal liver. 

### 2.5. Post-Treatment Image Assessment 

In ^18^F-FCH-positive PET/CT scans, standardized uptake values (SUVs) were calculated using regions of interest (ROI). In each patient, up to 3 of the most ^18^F-FCH-avid hepatic localizations were selected as the target lesions and the normal adjacent parenchyma as the background control. In order to normalize tumor SUVs, the ratio of SUVmax of the lesions to the mean SUV of the normal adjacent parenchyma (SUVmean), the tumor-to-normal liver ratio (TNR), was gauged. In order to minimize potential partial volume effects, the reference ROI in the normal hepatic parenchyma was drawn with a diameter of 2 cm. Eight weeks after the ^90^Y-TARE, patients underwent a further ^18^F-FCH PET/CT scan to assess metabolic response to ^90^Y-microspheres. 

Since no standard quantitative criteria have been established to define metabolic response on ^18^F-FCH PET/CT, the authors adopted the following: post-treatment PET/CT scans were compared with the pre-treatment ones and the relative change in TNR ratio (ΔTNR) was determined. Metabolic response was defined as a reduction of ≥50% in ΔTNR, while subjects were classified as non-responders in case of ΔTNR reduction < 50% or if new lesions were evident on the post-treatment PET/CT scan. 

In ^18^F-FDG-positive HCCs, SUV measurement was determined on the pre-treatment and 8-weeks post-treatment PET/CT scan using PET VCAR (GE Healthcare, Milwaukee, WI, USA). To assess metabolic response to ^90^Y-TARE, the follow-up PET/CT was compared to the pre-treatment scan according to the PET Response Criteria in Solid Tumors (PERCIST) [17].

### 2.6. Toxicity and Follow-Up

Toxicity was assessed according to the Common Terminology Criteria for Adverse Events (CTCAE), version 5.0, on the basis of laboratory tests, CT or PET/CT imaging, and clinical examinations. Before the procedure, all patients were submitted to laboratory tests, including total bilirubin, alanine transaminase, aspartate transaminase, alkaline phosphatase, and γ-glutamyl transpeptidase. After ^90^Y-TARE, all patients resumed a routine schedule of laboratory tests that were carried out at 2 and 4 weeks after the procedure and repeated at a 3-month interval. Clinical toxicities, including pain, fever, fatigue, and gastrointestinal adverse events, were evaluated at the regular follow-up visits.

### 2.7. Statistics

The normality of the distribution of the continuous variables was evaluated with the Shapiro–Wilk test. In the case of symmetric distribution, the variables are expressed with median, mean, and standard deviation (SD), while categorical data are represented as numbers and percentages.

Free survival (PFS) and overall survival (OS) were calculated by the Kaplan–Meier method (MedCalc 11.3.8.0; MedCalc Software, Mariakerke, Belgium), defined as the time from first ^90^Y-TARE to disease progression and to patient death, respectively. Fisher’s exact test was applied to examine differences in response to ^90^Y-TARE and PET/CT’s impact on clinical management among ^18^F-FCH-positive and ^18^F-FCH-negative/^18^F-FDG-positive patients. The Kaplan–Meier method was used to analyze differences in OS, and Cox regression analysis was applied to identify prognostic factors. Significance was established at two-tailed *p* < 0.05 level. 

## 3. Results

**Figure 1:**
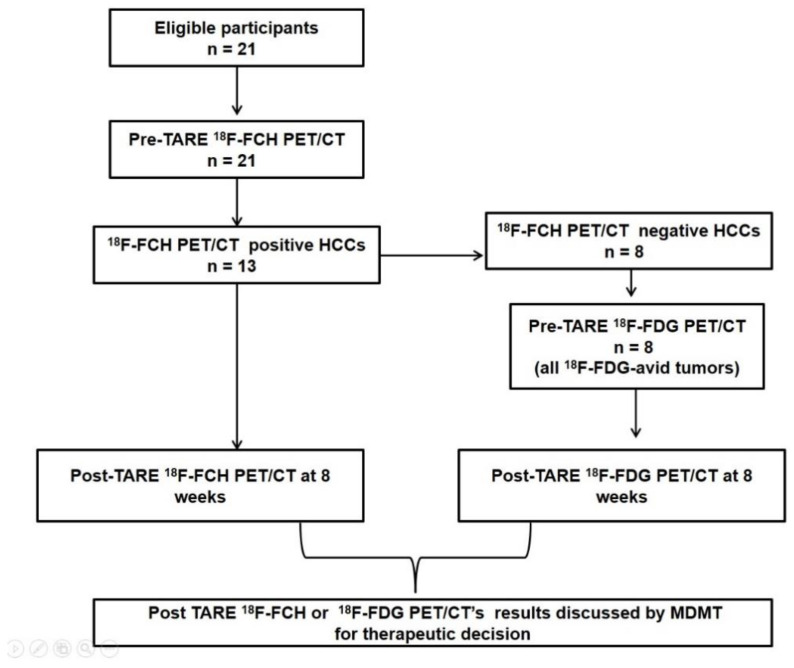
Diagnostic flow chart illustrating study design.

The interrogation of our database identified 21 HCC patients fulfilling inclusion criteria. All the included subjects were submitted between January 2018 and March 2019 to PET/CT with ^18^F-FCH or, in case of ^18^F-FCH-negative tumors, with ^18^F-FDG before and 8 weeks after ^90^Y-TARE, as shown by Figure 1. Clinical–demographic characteristics of the patients and the values of clinical and PET-derived quantitative variables are summarized in Table 1.

All patients had preserved Eastern Cooperative Oncology Group Performance Status (ECOG ≤ 1) and hepatic function (Child–Pugh score ≤ 6). Only two patients had extrahepatic metastases before ^90^Y-TARE enrollment: one subject showed a small lung nodule (1 cm diameter) stable in several repeated CT controls and one presented a small peritoneal localization to the anterior abdominal wall (1.5 cm diameter).

### 3.1. Pre-TARE PET/CT Imaging 

Thirteen of 21 patients (61.9%) presented ^18^F-FCH-positive HCC tumors on the pre-treatment PET/CT scan, while eight (38.1%) subjects were negative. ^18^F-FCH-positive PET/CT scans showed a median SUVmax of 16.5 and a mean SUmax of 15.5 ± 3.6, while the median and mean tumor-to-normal liver ratio (TNR) resulted in 2.2 and 2.2 ± 0.4, respectively. Notably, the two patients with known extrahepatic localizations before ^90^Y-TARE enrollment showed ^18^F-FCH incorporation in both HCC and metastases. 

In the eight patients with pre-treatment ^18^F-FCH-negative PET/CT scans, ^18^F-FDG PET/CT resulted positive in all cases, with a median SUVmax of 15.5 and a mean SUVmax of 14.3 ± 7.1.

Table 2 shows the correlation between PET/CT’s results and histological findings in nine patients, who had been submitted to surgery (*n* = 4) or biopsy (*n* = 5), classified according to World Health Organization (WHO) [18].

### 3.2. ^90^Y-TARE Procedure

An overall number of 21 ^90^Y-TARE procedures were performed, 17 were carried out according to a lobar ^90^Y-microsphere administration (i.e., 14 to the right hepatic lobe and 3 to the left lobe) and 4 following a sequential lobar approach. The mean administered activity was 1.5 ± 0.18. In 8 patients, a second TARE procedure was carried out on the basis of post-treatment PET/CT results. 

### 3.3. PET/CT Post-Treatment Assessment of Response

Thirteen patients (61.9%) showed metabolic response to ^90^Y-TARE. Of the 13 patients with pre-TARE ^18^F-FCH-positive PET/CT, ten (76.9%) exhibited metabolic response to ^90^Y-TARE with a mean ΔTNR of 68 ± 5.3. The two patients with ^18^F-FCH-positive extrahepatic localizations on pre-treatment PET/CT were both responders to ^90^Y-TARE with metastases’ stability on follow-up PET/CT scan. Three subjects with ^18^F-FCH-avid HCCs were non-responders: one patient showed ΔTNR < 50% and was categorized as stable metabolic disease, while other two subjects presented new-onset hepatic lesions and were therefore classified as progressive metabolic disease.

**Figure 2:**
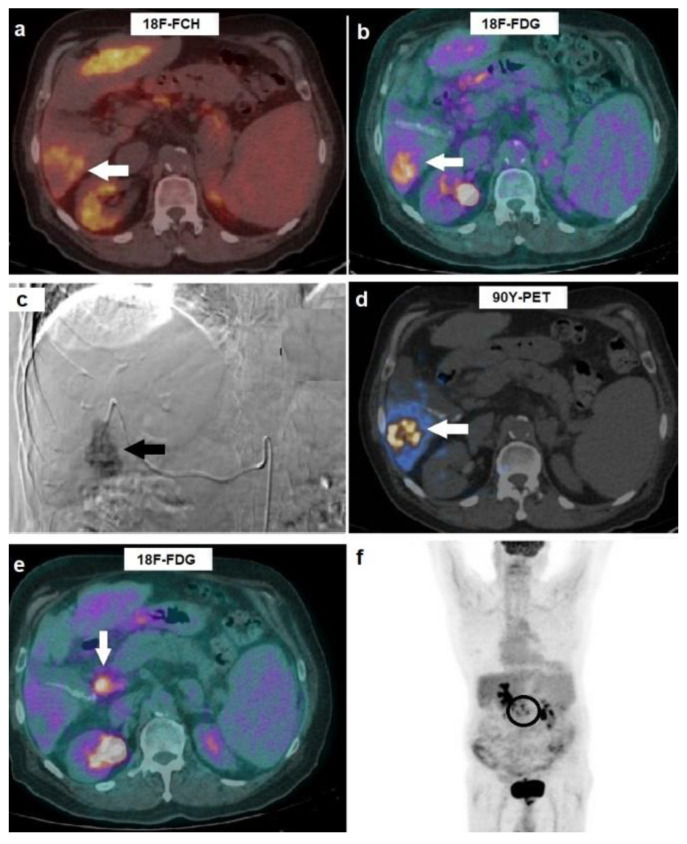
A 79-year-old man affected by HCV-related cirrhosis and HCC in the right hepatic lobe, previously treated with TACE and relapsed, referred to our center for ^90^Y-TARE. (**a**) Pre-treatment ^18^F-FCH PET/CT resulted negative for tracer incorporation in the HCC lesion located in the sixth hepatic segment (arrow). (**b**) Subsequently performed ^18^F-FDG PET/CT showed highly increased uptake (SUVmax 7.6) in HCC (arrow). (**c**) Pre-TARE angiogram, acquired after selective catheterization of the arterial branch for the sixth segment, clearly showed the hypervascularized lesion (arrow). (**d**) ^90^Y-PET, performed 4 h after ^90^Y-TARE, depicted selective microsphere accumulation in treated tumor (arrow). (**e**) On the 8-weeks post-treatment PET/CT scan, new-onset hypermetabolic pathological celiac adenopathies (arrow), in spite of complete regression of the treated HCC, were evident. (**f**) Maximum intensity projection ^18^F-FDG PET demonstrated well the metastatic localizations to abdominal nodes (circle).

Among the eight patients with pre-treatment ^18^F-FCH-negative/^18^F-FDG-positive HCCs, three (37.5%) subjects showed metabolic response to ^90^Y-TARE (partial metabolic response). Five patients were classified as non-responders: two subjects had stable metabolic disease, while three patients showed extrahepatic spreading with localizations to abdominal lymph nodes, as shown in Figure 2. 

Although the percentage of responders was higher in the ^18^F-FCH-positive patients with respect to ^18^F-FDG-positive ones (i.e., 76.9% vs. 37.5%), this difference did not reach the threshold of statistical significance (i.e., *p* = 0.46).

### 3.4. Post-Treatment PET/CT’s Impact on Patients’ Clinical Management

The results of post-treatment PET/CT evaluation at 8 weeks were discussed and analyzed by the multidisciplinary disease management team (MDMT), including nuclear medicine physicians and interventional radiologists together with each referring physician, who jointly defined the most appropriate therapeutic pathways, according to patients’ clinical status and eventual ^90^Y-TARE-related toxicity. 

**Figure 3:**
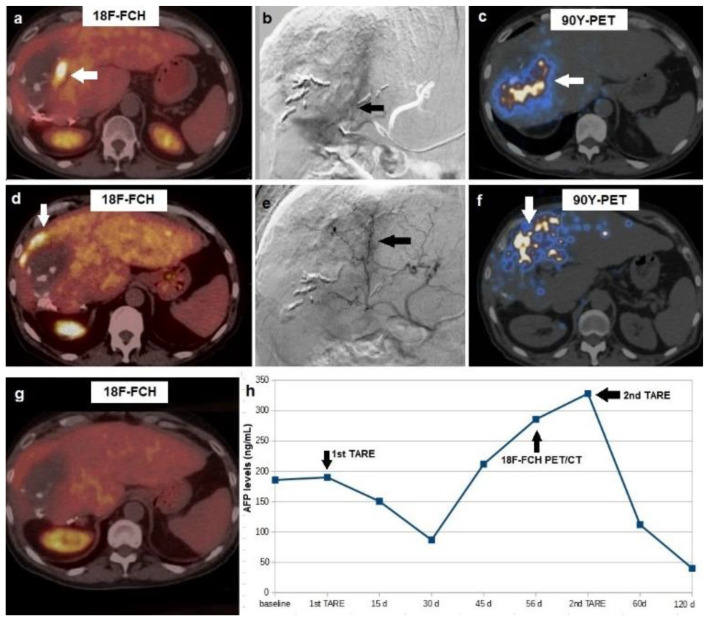
A 57-year-old male, affected by HCV-related cirrhosis and HCC in the right hepatic lobe, previously treated with laser ablation and surgical resection, referred to our center for ^90^Y-TARE. (**a**) Pre-treatment ^18^F-choline PET/CT demonstrated increased tracer incorporation in the infero-medial portion of the HCC lesion (arrow), which showed a central necrotic, hypodense area at co-registered CT slice. (**b**) Pre-TARE angiogram, performed after selective catheterization of the arterial feeder of the active tumor tissue detected by PET/CT, showed the hypervascularized tumor (arrow). (**c**) ^90^Y-PET acquired 4 h after TARE demonstrated ^90^Y-microsphere deposition in the treated tumor (arrow). (**d**) At 8-weeks post TARE PET/CT, a new-onset metabolically active HCC tissue was detected in the superior rim of the lesion (arrow), in spite of complete response of the tumor tissue previously located in HCC’s infero-medial portion. Therapeutic decision was to carry out a second ^90^Y-TARE: (**e**) a new angiogram was acquired after selective catheterization of the arterial branch for the upper hepatic segments (arrow). (**f**) ^90^Y-PET at 4 h post TARE demonstrated ^90^Y-microsphere accumulation in the superior portion of the HCC lesion. (**g**) Post-treatment PET/CT at 8 weeks after the second ^90^Y-TARE showed complete metabolic response. (**h**) Graph representation of alphafetoprotein trend during the different phases of the patient’s clinical management. Overall survival was 25 months.

Post-TARE PET/CT affected patients’ clinical management in 10 out of 21 cases (47.6%). In particular, a second ^90^Y-TARE treatment was performed in eight patients with evidence of metabolically active HCC remnant (*n* = 7) or new-onset lesion (*n* = 1). In such cases, a further angiography was performed before the second ^90^Y-TARE, and vascular imaging was accurately examined in order to identify and selectively catheterize the arterial branch supplying the metabolically active tissue disclosed by ^18^F-FCH (*n* = 7) or ^18^F-FDG (*n* = 1) PET-imaging. In all these patients, complete metabolic response was registered after PET-guided ^90^Y-TARE (Figure 3).

**Figure 4:**
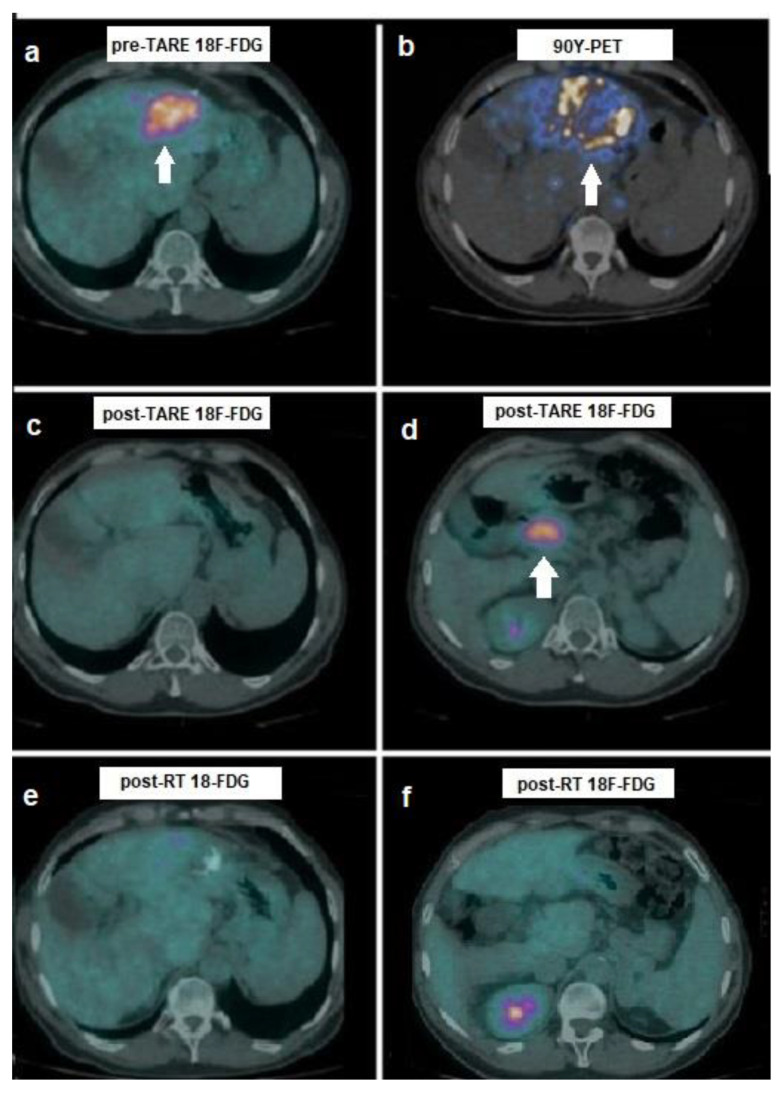
A 51-year-old affected by HCV-related cirrhosis and HCC in the left hepatic lobe, with portal vein invasion, progressive during systemic therapy with sorafenib. On pre-treatment examination, HCC resulted ^18^F-FCH-negative, but (**a**) intensely positive on ^18^F-FDG PET/CT (arrow, SUVmax 17.7). (**b**) After selective catheterization of HCC’s arterial feeder, he was administered with ^90^Y-microspheres: ^90^Y-PET showed a peripheral pattern of accumulation with respect to HCC lesion (arrow). At 8-weeks post-treatment ^18^F-FDG PET/CT, a complete regression of HCC in the left lobe was evident (**c**), although the onset of an intensely hypermetabolic celiac lymph adenopathy was detected ((**d**), arrow). Therapeutic decision was stereotactic RT on the ^18^F-FDG-positive metastasis. On follow-up ^18^F-FDG PET/CT carried out at 2 months after RT, complete metabolic response was registered both in the hepatic lesion (**e**) and in nodal metastasis (**f**). Overall survival resulted in 36 months.

PET/CT meaningfully affected two out of three patients with evidence of extrahepatic progression on post-treatment ^18^F-FDG PET/CT. In such cases, stereotactic RT was carried out on metastatic localizations to celiac lymph nodes: post-treatment PET/CT images were utilized to draw biological target volume (BTV) that was incorporated into radiation therapy (RT) planning with optimal clinical and imaging response in both cases, as shown in Figure 4.

In 11 patients, post-treatment PET/CT impact was scored as non-relevant. In particular, monitoring patients through periodic examinations until the evidence of progressive disease was the clinical decision in six patients with partial metabolic response (^18^F-FCH-positive, *n* = 4 and ^18^F-FDG-positive PET/CT, *n* = 2) and in three patients with stable metabolic disease on post-TARE (^18^F-FDG-avid, *n* = 2 and ^18^F-FCH-avid, *n* = 1). In such cases, a further ^90^Y-TARE was not carried out, in spite of metabolically active HCC remnant detected on post-treatment PET/CT, due to increased value of bilirubin (grade II, *n* = 1) or hepatic enzymes (grade II, *n* = 2), ascites (grade I, *n* = 1), or since subjects (*n* = 5, in all cases aged > 69 years) refused a repeated ^90^Y-microsphere administration due to post-embolization syndrome (PES), mainly consisting of nausea and vomiting, occurred during the first 72 h following the first ^90^Y-TARE procedure.

In two subjects with progressive metabolic disease, the therapeutic decision was the implementation of tyrosine-kinase therapy.

PET/CTs affected 7 out of 13 (53.8%) ^18^F-FCH-positive and 3 out of 8 (37.5%) ^18^F-FDG-positive tumors, as shown in Table 3, without significant difference among the two groups (*p* = 0.65).

### 3.5. Prognostic Factors on Patient Survival 

The mean PFS and OS in all patients were 9.3 ± 2.1 months (95% confidence interval, 5.1–13.4 months; median 8 months) and 18.6 ± 2.1 months (95% confidence interval, 14.3–22.9 months; median 18 months), respectively. In order to perform Kaplan–Meier analysis for OS, continuous variables (i.e., bilirubin levels, age) were dichotomized by median value, while categorical data were dichotomized, as indicated in Table 4. Alphafetoprotein levels were not considered in the analysis, since they were found increased only in 11 patients. 

**Figure 5:**
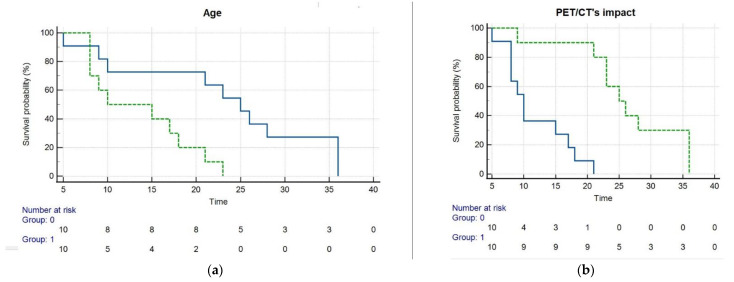
Kaplan–Meier analysis depicts overall survival (months) after ^90^Y-TARE as a function of patient age (**a**) and post-TARE PET/CT’s impact on clinical management (**b**). Panel a shows that patients with age ≤ 69 years (blue line, group 0) had significantly (*p* = 0.005) longer OS than those aged > 69 years (green line, group 1). Panel b demonstrates that patients whose clinical management was influenced by PET/CT (green line, group 1) had meaningfully (*p* < 0.001) longer OS than those in which PET/CT‘s impact was scored as non-relevant (blue line, group 0).

By Kaplan–Meier analysis (Figure 5), patients whose clinical management was influenced by post-treatment PET/CT had a significantly (*p* < 0.001) longer OS (26.3 ± 2.6 months) than those in which PET/CT’s impact was scored as non-relevant (11.2 ± 1.5 months). Furthermore, subjects with age ≤ 69 years exhibited a significantly (*p* = 0.005) longer OS (23.1 ± 3.3 months) than older patients (13.7 ± 1.8 months).

In Cox multivariate analysis, including age, sex, uninodular vs. plurinodular disease, bilirubin levels, tumor burden, cirrhosis, previous therapies, presence of metastases, portal vein invasion, or metabolic response, post-TARE PET/CT’s impact on clinical management remained the only significant predictor of OS (*p* = 0.01, hazard ratio = 0.01, 95% confidence interval, 0.0007–0.43).

## 4. Discussion

Our real-world study assessed the clinical impact of post-treatment PET/CT with ^18^F-FCH or ^18^F-FDG in HCC patients submitted to ^90^Y-TARE. We found that a PET/CT evaluation at 8-weeks post-treatment influenced clinical management in 47.6% of subjects, through the implementation of PET-directed therapies. In addition, post-TARE PET/CT’s impact on clinical management resulted in a significant predictor of patients’ final outcome both by Kaplan–Meier and Cox multivariate analysis. 

PET/CT is a well-established imaging modality in oncology and plays an essential role for staging and monitoring response to treatment in many oncological conditions. Nevertheless, metabolic imaging is not routinely considered in HCC diagnostic work-flow. In a comparative study performed by Talbot et al. [19] in 81 patients with suspected liver nodules, PET/CT with ^18^F-FCH showed a significantly higher sensitivity than that with ^18^F-FDG (88% vs. 68%, *p* = 0.07) for HCC diagnosis, although ^18^F-FDG had a higher detection rate for the less differentiated and more aggressive forms. ^18^F-FDG incorporation into HCC is not only correlated with high histological grade, but also with the expression of genes strictly linked with cell survival, cell-to-cell adhesion, or cell spreading [20]. The combined use of ^18^F-FCH and ^18^F-FDG has been proposed for HCC imaging through PET technology according to the grade of differentiation: in a large cohort (*n* = 177) of HCC patients, dual tracer PET/CT substantially affected staging according to the Barcelona Clinical Liver Cancer (BCLC) classification and consequently changed subjects’ management [21]. 

Few studies investigated the role of metabolic response assessed by PET/CT in HCC patients treated with ^90^Y-microspheres. In a previous report from our group [22], we found decreased total lesion glycolysis (TLG), measured at 1 month after ^90^Y-TARE, associated with a trend toward a longer OS in poorly differentiated HCC with portal vein invasion. Hartenbach and colleagues employed PET/CT with ^18^F-fluoroethylcholine in 24 patients with locally advanced HCC and initially elevated AFP level; from semiquantitative analysis, increased SUV mean at diagnosis, decreased SUV max and in tumor-to-background ratio after ^90^Y-therapy (i.e., Δmaximum SUV and Δtumor-to-background ratio, respectively) showed the highest area under the curve to predict patient response [23]. The results reported by Hartenbach’s group are substantially in agreement with those more recently described by Aujay and coworkers in nine HCC patients treated with ^90^Y-TARE and monitored through ^18^F-FCH PET/CT [24]. 

Reizine and colleagues applied dual tracer PET/CT with ^18^F-FCH and ^18^F-FDG in 37 HCC patients submitted to ^90^Y-TARE for the assessment of early post-treatment response at 4–8 weeks after procedure [13]. All of the enrolled subjects were submitted to dual tracer PET/CT before ^90^Y-microsphere administration: 28 patients resulted ^18^F-FDG-positive; 9 were ^18^F-FCH positive. Metabolic response detected on early post-treatment PET/CT showed 100% sensitivity and specificity for predicting 6-month radiological response assessed by mRECIST; furthermore, metabolic response was a significant predictor of OS. To the best of our knowledge, our report is the first study specifically highlighting the clinical usefulness of PET/CT with ^18^F-FCH or ^18^F-FDG after ^90^Y-TARE in order to promptly identify patients with residual or progressive metabolically active disease, amenable to timely PET-guided retreatments. Of note, our results, indicating the feasibility of repeated ^90^Y-TARE to treat recurrent or residual primary disease in similar hepatic arterial lobe or segments, are substantially in line with the terms of safety and efficacy for ^90^Y-loaded glass microspheres, as published by Badar and colleagues [25]. 

The optimal time point to assess the radiobiological effects of ^90^Y-microspheres has yet to be defined. It is expected that ^90^Y-TARE might also have delayed effects, although this assumption is mainly based on the limitations of traditional imaging techniques in the early assessment of response to ^90^Y-TARE. In a recently published in vitro study, in fact, ^90^Y-microspheres were found to reduce colorectal cancer cell proliferation as early as within 96 h of observation [26]. In this perspective, a time interval of 8 weeks after procedure might represent a reasonable gap to assess ^90^Y-microsphere effects on HCC. 

As far as it concerns the role of patients’ age in HCC treated with ^90^Y-TARE, our data are not in line with those reported by the retrospective study conducted by The European Network on Radioembolization with Yttrium-90 resin microspheres study group (ENRY), including elderly (≥70 years, *n* = 128) and younger (<70 years, *n* = 197) subjects [27]. In the cited paper, ^90^Y-TARE was equally tolerated in both cohorts with no significant differences in survival between the two groups. In our study, the effect of age on patients’ final outcome might be explained by the higher compliance registered in younger subjects to be submitted to further PET-directed therapy (second ^90^Y-microsphere administration or RT) for eradicating HCC remnant or new-onset metastases. 

In our real-world study, we employed ^18^F-FCH as the first line tracer for pre-treatment imaging of HCC with the aim of identifying the metabolically active tissue to be targeted with ^90^Y-microsphere administration. It is worth mentioning that we did not systematically perform dual tracer PET/CT in all of the enrolled patients; only subjects with ^18^F-FCH-negative tumors were submitted to ^18^F-FDG as a second-line functional imaging modality, in order to limit the radiation burden delivered to patients. We cannot exclude that some of the ^18^F-FCH-positive tumors might also express a variable grade of ^18^F-FDG-avidity. 

^90^Y-microspheres are recommended and generally employed in subjects with advanced HCC, often heavily pre-treated, progressing after surgery, TACE, or systemic therapy [28]. In such cases, liver anatomy may be altered by the previously performed treatments, and identifying HCC-viable tissue might be challenging on conventional morphological imaging (ce-CT or MRI). In these patients, PET/CT with ^18^F-FCH or ^18^F-FDG may have an important role supporting clinicians both during ^90^Y-TARE planning and in response assessment. 

Our study has several limitations. First of all, the limited number of included patients and its retrospective nature may have introduced a selection bias in patient enrollment. However, it has to be underlined that our sample size (*n* = 21), although small, is not significantly different with respect to that included in the other cited papers focusing on ^18^F-FCH PET/CT on HCC submitted to ^90^Y-TARE (i.e., Hartenbach et al., *n* = 24; Reizine et al., *n* = 37) [13,23]. 

Furthermore, since our retrospective real-life world study was mainly aimed to provide information on the use of PET/CT with ^18^F-FCH or ^18^F-FDG in a specific HCC clinical setting, we did not perform a comparative assessment of ^18^F-FCH/^18^F-FDG PET/CT’s impact on patient management with respect to that of more conventional diagnostic techniques, such as ce-CT and MRI [29]. In this regard, a prospective study performed by Barabasch and coworkers in 36 consecutive patients with liver metastases (20 colorectal, 14 breast cancer, 2 with other malignancies), submitted to ^18^F-FDG PET/CT and diffusion-weighted MRI (DWI-MRI) before and 4–6 weeks after ^90^Y-TARE, showed that response based on DWI-MRI outperformed PET/CT for predicting final outcome [30]. Nevertheless, MRI-DWI’s impact on clinical management in HCC subjects treated with ^90^Y-microspheres, as compared to that of PET/CT, has not yet been assessed. This topic is worthy of future investigations. 

In addition, we used the BSA method for the calculation of ^90^Y-microsphere prescribed activity, while personalized provisional dosimetry is now recommended as the state-of-the-art to determine the dose delivered to tumor and non-tumor parenchyma [31]. In a prospective study, Ho et al. [32] employed dual tracer PET/CT with ^18^F-FDG and ^11^C-acetate, another surrogate imaging biomarker of phospholipid synthesis, to define the relationship between tumor dose (TD) and response, according to HCCs’ grade of differentiation. In agreement with our findings, the authors found a higher response rate in more differentiated (i.e., ^11^C-acetate-positive) than in aggressive (^18^F-FDG-positive) HCCs (72.4% vs. 25%, respectively); furthermore, TD for response resulted meaningfully higher for poorly differentiated with respect to well/moderately differentiated HCCs (262 Gy vs. 152/174 Gy). The role of dual tracer PET/CT for the personalized dose prescription in HCC submitted to ^90^Y-TARE will be topic of future investigations. 

## 5. Conclusions

In our real-world study, post-treatment PET/CT with ^18^F-FCH or ^18^F-FDG, carried out at 8 weeks after ^90^Y-TARE, influenced patients’ clinical management by the implementation of PET-guided therapies and significantly affected final outcome. Further well-designed studies with larger cohorts, ideally prospective and entailing multicenter co-operations, are needed to better define the role of metabolic imaging in this specific clinical setting. 

## Figures and Tables

**Table 1:** Patients’ baseline clinical–demographic features and results of quantitative parameters.

Age (Years)		-
median	69	
mean ± SD	66.3 ± 9.2	
Sex	*n*	%
male	18	85.7%
female	3	14.3%
HBV/HCV infection	*n*	%
positive	19	90.5%
negative	2	9.5%
Previous Therapies	*n*	%
RFA and/or TACE	15	71.4%
surgery	4	19%
sorafenib	6	28.5%
Portal Vein Invasion	*n*	%
present	11	52.4%
no evidence	10	47.6%
Extrahepatic Metastasis	*n*	%
present	2	9.5%
no evidence	19	90.5%
HCC Presentation	*n*	%
uninodular	17	81%
plurinodular	4	19%
AFP levels	*n*	%
increased	11	52.4%
not increased	10	47.6%
Baseline levels of bilirubin (mg/dL)	mean ± sd	median
	1.4 ± 0.2	1.4
Tumor burden on baseline PET/CT	*n*	%
<25	16	76.2%
>25	5	23.8%
^18^F-FCH PET-derived parameters	mean ± sd	median
SUVmax	15.5 ± 3.6	16.5
TNR	2.2 ± 0.4	2.2
^18^F-FDG PET-derived parameters	mean ± sd	median
SUVmax	14.3 ± 7.1	15.5

Abbreviations: HBV—hepatitis virus B infection; HCV—hepatitis virus C infection RFA—radiofrequency ablation; TACE—transarterial chemoembolization, HCC—hepatocellular carcinoma; AFP—alphafetoprotein; TNR—tumor-to-normal liver ratio; SUVmax—maximum standardized uptake value.

**Table 2:** Correlation between the results of ^18^F-FCH/^18^F-FDG PET/CT and histological findings in 9 HCC patients previously submitted to biopsy or surgery.

Histological Grading	^18^F-FCH+	^18^F-FCH−/^18^F-FDG+
Well differentiated	5	-
Moderately differentiated	1	2
Poorly differentiated	-	1

**Table 3:** Impact of ^18^F-FCH/^18^F-FDG PET/CT on patient management.

Therapeutic Impact	^18^F-FCH+	^18^F-FCH−/^18^F-FDG+
PET-guided second ^90^Y-TARE	7	1
PET-guided RT	-	2
No relevant impact	6	5

**Table 4:** Results of Kaplan–Meier analysis of variable potentially predicting OS.

	No. of Patients	Overall Survival (mo), Mean (95% CI)	*p*
Sex			
male	18	17.6 (13.0–22.2)	0.39
female	3	25.0 (14.0–35.9)	
Age			
≤69 y	11	23.1 (16.6–29.7)	0.005 *
>69 y	10	13.7 (10.0–17.3)	
Bilirubin levels			
≤1.4 mg/dL	12	18.8 (12.9–24.7)	0.95
>1.4 mg/dL	9	18.4 (11.8–25)	
Cirrhosis			
no	2	21.5 (8.7–34.2)	0.83
yes	19	18.3 (13.7–23)	
Previous TACE-RFA			
no	6	17.6 (8.3–25.9)	0.99
yes	15	19 (14.1–23.9)	
Previous Surgery			
no	17	18 (12.7–23.2)	0.98
yes	4	21.5 (18.1–24.8)	
Previous Sorafenib			
no	15	16.2 (11.8–20.5)	0.08
yes	6	24.8 (15.5–34.1)	
Extrahepatic Metastases			
no	19	18.4 (13.8–23.1)	0.97
yes	2	20.5 (14.7–28.3)	
Portal Vein Invasion			
no	10	15.5 (10.8–20.1)	0.12
yes	11	21.5 (6.6–15.3)	
Uni- vs. Plurinodular			
pluri	4	16.2 (7.7–24.7)	0.54
uni	17	19.2 (14.2–24.2)	
Metabolic response			
no	8	13.6 (6.0–21.1)	0.15
yes	13	21.7 (17.1–26.3)	
PET/CT’s impact on clinical management			
no	11	11.7 (8.5–14.7)	<0.001 *
yes	10	26.3 (21.0–31.5)	

* statistical analysis resulted in significant *p*.

## Data Availability

Datasets are available upon request.
